# Heterogenous within-herd seroprevalence against epizootic hemorrhagic disease virus type 8 (EHDV-8) after massive virus circulation in cattle in France, 2023

**DOI:** 10.3389/fvets.2025.1562883

**Published:** 2025-05-14

**Authors:** Corinne Anthonioz, Yann Abadie, Elodie Reversat, Annie Lafargue, Manon Delalande, Thierry Renaudineau, Laurent Delobel, Nathalie Verdeille, David Ngwa-Mbot, Kristel Gache, Emmanuel Garin, Fabien Corbiere

**Affiliations:** ^1^Groupement de Défense Sanitaire (Animal Health Farmers' Organization) du Tarn et Garonne, Montauban, France; ^2^Groupement de Défense Sanitaire (Animal Health Farmers' Organization) des Hautes-Pyrénées, Tarbes, France; ^3^Groupement de Défense Sanitaire (Animal Health Farmers' Organization) de l’Ariège, Foix, France; ^4^Groupement de Défense Sanitaire (Animal Health Farmers' Organization) du Lot-et-Garonne, Agen, France; ^5^Groupement de Défense Sanitaire (Animal Health Farmers' Organization) des Deux-Sèvres, Vouillé, France; ^6^Groupement de Défense Sanitaire (Animal Health Farmers' Organization) de la Vendée, La Roche sur Yon, France; ^7^Groupement de Défense Sanitaire (Animal Health Farmers' Organization) de la Loire-Atlantique, Nantes, France; ^8^Groupement de Défense Sanitaire (Animal Health Farmers' Organization) du Tarn, Albi, France; ^9^GDS France (National Animal Health Farmers’ Organization), Paris, France; ^10^UMR INRAE-ENVT 1225 IHAP, Toulouse, France

**Keywords:** cattle, epizootic hemorrhagic disease virus type 8, epidemiology, seroprevalence, immunity, EHDV-8

## Abstract

**Background and objectives:**

The emergence of Epizootic Hemorrhagic Disease Virus-8 (EHDV-8) in mainland France in 2023 led to thousands of clinical outbreaks in cattle herds and likely led to the natural immunization of a large number of animals. However, uncertainties persist regarding the extent of this immunity, both within herds and across affected regions. This study therefore aimed at investigating the variability of within-herd seroprevalence in clinically affected and non-affected herds across geographical areas with differing levels of disease incidence.

**Methodology:**

A study was launched in February 2024 to assess the variability of within-herd seroprevalence in three geographical areas with varying EHDV-8 clinical incidence. A total of 2,763 serums samples from cattle over 24 months in 30 herds with clinical outbreaks and 31 herds without reported clinical case were analyzed using a commercial competitive ELISA.

**Results:**

A strong south–north seroprevalence gradient was observed, with the highest animal-level seroprevalence evidenced in the southernmost zone (Pyrenean Piémont) (82.6%, CI 95%: 81.1–83.9), which also experienced the highest incidence of clinical outbreaks. In contrast, significantly lower seroprevalence levels were found in the more northern areas: (zone 2: 11.6, 95% CI: 10.7–12.7; zone 3: 0.3, 95% CI: 0.1–1.1), where clinical outbreaks were less frequent. The within-herd seroprevalence varied widely among herds but was significantly higher in those located in the southernmost zone, compared to other areas. Within each zone, no significant differences in seroprevalence were observed between clinical outbreak herds and non-outbreak herds.

**Discussion:**

This study highlights significant geographic and between herd variability in seroprevalence against EHDV-8 after the major virus circulation experienced in 2023, and provides critical insights into regional risks and the potential impact of future EHDV-8 circulation.

## 1 Introduction

A variety of anthropogenic factors, including climate change and global trade are increasing the risk of emerging viral diseases, among which those transmitted by arthropod insects, in previously non-endemic territories. In ruminants, a recent example is the emergence in Europe of several Orbiviroses, including bluetongue in the 1950s (1) and, more recently, Schmallenberg virus disease (2), and epizootic hemorrhagic disease (EHD) (3, 4).

EHDV is an Orbivirus related to the bluetongue virus (BTV) and shares a variety of domestic and wild ruminant hosts, including cattle, sheep, and white-tailed deer. The associated morbidity and mortality are highly variable depending on the species, individuals, and viral serotypes involved. Currently, 7 serotypes of EHDV are recognized (EHDV-1, EHDV-2, EHDV-4 to 8) and two additional putative serotypes (EHDV-9 and EDHV-10) have been reported (5, 6). EHDV is known to cause a severe disease in white-tailed deer and generally mild clinical signs in cattle (7). However, some episodes have been associated with increased pathogenicity, resulting in significant economic losses: Japan in the mid-20th century (Ibaraki EHDV-2 virus), Reunion Island (2003), Israel (2006 and 2015), Morocco, Algeria and Jordan (2006), Turkey (2007), China and USA (2013). During these episodes, serotypes 2, 6 and 7 were predominantly identified. In many countries (USA, Australia, China, Japan…), several serotypes circulate, without consistently leading to clinical outbreaks.

In 2021, EHDV-8 was unexpectedly isolated in Tunisia and caused more than 200 clinical outbreaks (8) before it was identified for the first time on the European continent in October and November 2022 in Sardinia, Sicily and southern Spain (3, 4, 9). In September 2023, the first case was identified in France in the Pyrénées-Atlantiques department, an administrative district bordering Spain on the French side of the Pyrenees (10).

In France, virus spread was extensive and rapid in the fall of 2023, with 3,729 confirmed clinical outbreaks reported in cattle as of 16.01.2024 and 4,310 as of 31.05.2024, mostly in the southwestern French departments bordering the Pyrenees. The incidence of clinical cases and associated mortality varied widely across farms (10). A halt in viral circulation was observed during winter 2023–2024, most likely due to a reduced abundance and activity of *Culicoides* vectors and increased extrinsic incubation period at cooler temperatures (11).

This first major circulation of EHDV-8 on mainland France has likely led to immunization of a large number of cattle, well beyond just those clinically affected. Although the duration of neutralizing antibodies against EHDV-8 in cattle has not been yet definitely established, studies on other EHDV variants or BTV suggest that this acquired immunity is likely to persist for several years, potentially mitigating clinical manifestations in the event of reinfection (12, 13). Herd-level acquired immunity may also offer a protective effect by limiting the extent of a new viral circulation.

However, several questions remain, particularly regarding the extent of this immunization within herds and across affected regions. In the absence of available vaccine against EHDV-8 in the early months of 2024, these unknowns have raised concerns about the consequences of resumption of viral circulation as early as summer 2024. Later, an effective vaccine against EHD received temporary authorization for use in France on August 2024. On September 2024, the French government established a vaccination zone to limit the disease’s spread towards eastern France by creating a vaccination belt along the regulated zone’s border.

The proposed study aimed to investigate the within-herd seroprevalence in geographical areas that have experienced clinical outbreaks of EHD to varying degrees in 2023. The expected results were (i) insight into potential immunity acquired following the late 2023 viral circulation, which could inform the consequences of vector activity resumption and viral circulation in 2024 (ii) an understanding of the relationship between within-herd seroprevalence and observed clinical incidence, by geographic region, to assess residual risk (i.e., proportion of immunized and non-immunized animals within a herd) based on clinical data. These results could provide useful information for geographical areas that have not yet experienced viral circulation.

## 2 Materials and methods

### 2.1 Geographic zones of investigation

This study was set-out in late February 2024. Three geographic zones were arbitrary defined according to the timing and magnitude of EHD clinical outbreak incidence, based on official data as at February, 15, 2024.

- The first zone, “Zone 1,” included the departments of Ariège (code 09) and Hautes-Pyrénées (code 65), where the number of EHD clinical outbreaks was the highest, with 264 and 691 reported clinical outbreaks, respectively. These departments were among the first affected in autumn 2023 (first detection dates: Hautes-Pyrenees on September 8, 2023, and Ariège on September 20, 2023).
- Zone 2 consisted of the departments of Tarn (code 81), Tarn-et-Garonne (code 82) and Lot et Garonne (code 47), were the disease was first detected between October 6 and 20, 2023 and with an intermediate number of clinical outbreaks (overall *n* = 41 as at February, 15, 2024).
- Zone 3, to the north, included the departments of Loire-Atlantique (code 44), Deux-Sèvres (code 79), and Vendée (code 85), where the disease was detected by the end of November 2023, with very few clinical outbreaks (*n* = 7) until Summer 2024.

### 2.2 Farm selection

**Figure 1:**
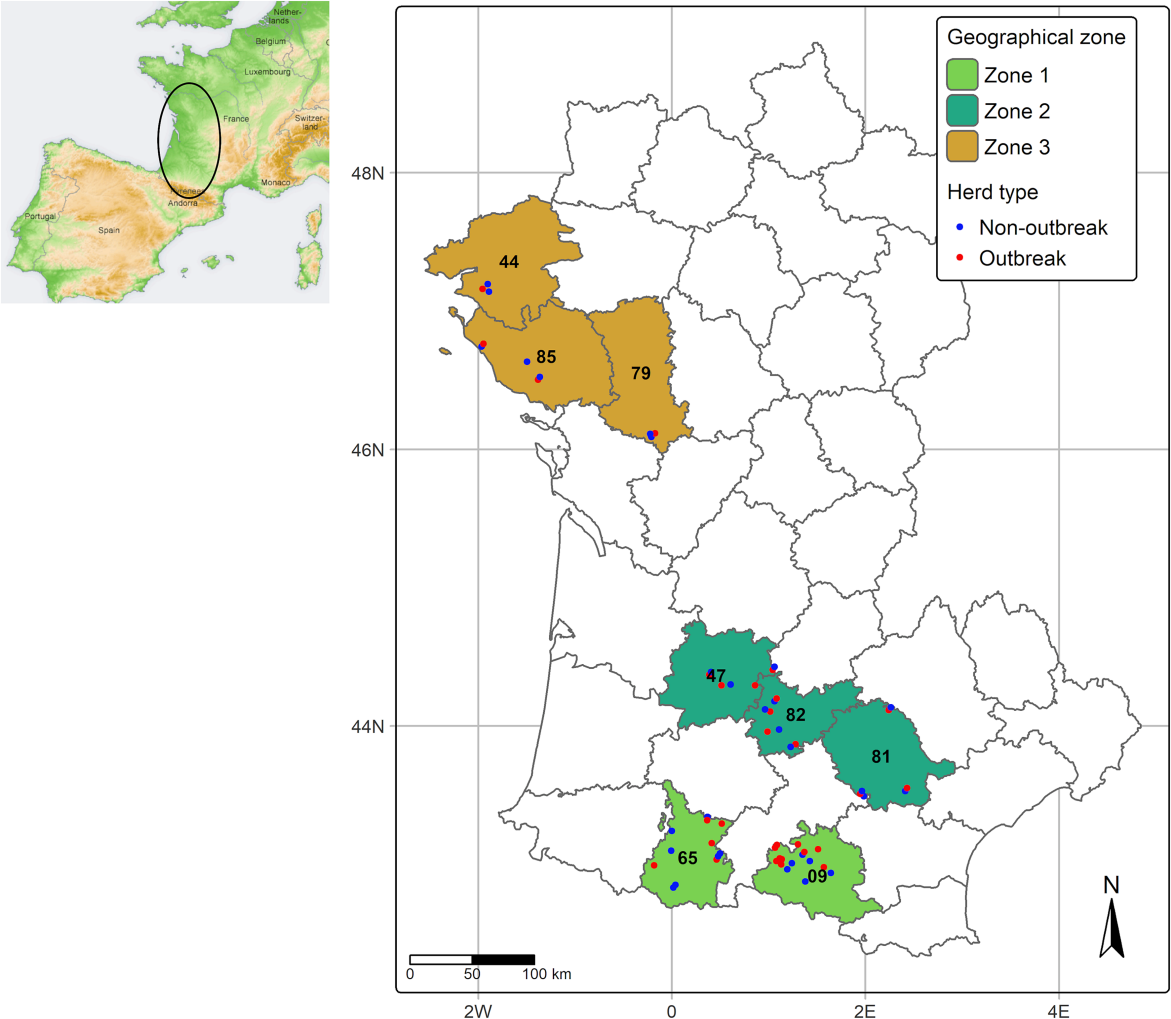
Geographical distribution of investigated geographical zones and herds. The position of some herds has been slightly shifted to avoid overlapping.

Within each geographical zone, “outbreak” herds were selected at random by the local Animal Health Farmers’ Organization (Groupement de Défense Sanitaire, GDS). All outbreak herds had at least one bovine exhibiting clinical signs consistent with EHD, which was subsequently confirmed through a positive result on EHDV RT-PCR conducted by certified local veterinary laboratories. “Non-outbreak” herds were required to have similar management practices and be located near the outbreak herds, within a 5 to 10 km radius. No cases of BTV-8 infection was evidenced in any investigated herd. The geographic distribution of herds included in the study, mapped at the level of communes (municipalities), is shown in Figure 1.

A total of 61 volunteer farms were enrolled in the study (30 outbreak herds and 31 non-outbreak herds), primarily beef herds, except for two dairy herds (non-outbreak) in the Hautes-Pyrénées department. The distribution of herds by geographic zone and herds characteristics are detailed in Table 1. Herd size did not significantly differ (Wilcoxon–Mann–Whitney test for ranks, *p* = 0.46) between outbreak and non-outbreak herds. However, herds in Zone 3 were significantly larger than those in the Zones 1 and 2 (*p* = 0.001), in accordance with what is observed in the general herd population.

**Table 1:** Herd characteristics.

Geographical zone	Number of herds			Number cattle > 24 months old	
	Outbreak	Non-outbreak	Total	Median	IQR*
Zone 1	15	13	28	61	50–93
Zone 2	11	11	22	52	36–60
Zone 3	4	7	11	125	89–201
Total	30	31	61	78	42–93

* IQR: inter-quartile range.

### 2.3 Selection of cattle within herds

Serological analyses were conducted by certified local veterinary laboratories on serum samples collected during the 2023–2024 mandatory annual surveillance program for brucellosis and infectious bovine rhinotracheitis (IBR) and stored frozen.

A sampling plan was proposed, defining the number of cattle to test based on the number of cattle over 24 months in each herd, to estimate within-herd seroprevalence with an absolute precision of 10%. The expected seroprevalence was set at 30% in Zones 2 and 3 and 80% in Zone 1. This resulted in the need to test between 30 and 60 animals for herds ranging in size from 40 to 200 cows over 24 months.

However, due to reduced cattle sampling following the relaxation of IBR prophylactic measures in France, a maximum of 40 serum samples was available for certain herds. In contrast, all cattle over 24 months old had been blood-sampled in some herds, resulting in unequal sampling rates and precision across the studied herds. The median sampling rate (proportion of cattle over 24 months included in the study sample) was 71.7% (1st quartile: 44.9%; 3rd quartile: 100%). In 26 of the 61 herds, blood samples from more than 90% of cattle over 24 months were included in the study sample. Sampling rates did not differ significantly between outbreak and non-outbreak herds (Wilcoxon–Mann–Whitney test for ranks, *p* = 0.77) but were higher in Zone 2 (median 100%) than in Zone 1 (median 65.8%) and Zone 3 (median 40.4%) (*p* < 10^−4^).

### 2.4 Blood sampling dates

In the included herds, blood sampling carried out within the annual prophylactic campaign had ranged from December 1, 2023, to May 23, 2024, with three-quarters of herds sampled between late December 2023 and mid-February 2024. For the 30 outbreak herds, the time interval between the first clinical cases reported by the farmer and blood sampling ranged from 1 to 6.5 months (median 3 months). For five herds, this interval was less than 50 days. Sampling dates did not significantly differ between outbreak and non-outbreak herds, either overall or within each geographic zone (overall, Wilcoxon–Mann–Whitney test for ranks, *p* = 0.6, Zone 1: *p* = 0.32; Zone 2: *p* = 0.74; Zone 3: *p* = 0.92). Similarly, no significant difference could be evidenced between geographical zones (Kruskal-Wallis test for ranks, *p* = 0.63). It is therefore unlikely that differences in sampling dates between outbreak and non-outbreak herds, or across geographical zones, could have biased the seroprevalence results due to variations in the time between exposure to virus circulation and blood sampling.

### 2.5 Serological analyses

Serological analyses were conducted by certified local veterinary laboratories on sera from cattle over 24 months of age, using a competitive ELISA kit (ID Screen^®^ EHDV Competition, Innovative Diagnostics, Grabels, France) (14). Analyses were performed and interpreted according to the manufacturers’ instructions. Samples with a competition percentage ≤ 30%, [30 – 35%], > 35% were considered positive, doubtful or negative, respectively. The sensitivity and specificity of this kit was considered perfect (100%) in subsequent analyses.

Out of the 2,763 sera tested, 22 samples (7.9%) from 5 outbreak herds and 6 non-outbreak herds were classified as doubtful by the ELISA manufacturers’ instructions and subsequently treated as positive in the analysis. This had a minimal effect on the interpretation of the results.

### 2.6 Others information

At the time of inclusion in the study, farmers were interviewed by phone call to collect specific information regarding the within herd EHDV outbreak: date of the first clinical case, overall number of cattle over 24 months old with clinical signs attributable to EHDV since the beginning of the outbreak, and among them, the number still present at the time of the annual prophylactic campaign.

### 2.7 Data analysis

The precision of apparent seroprevalence estimates varied among herds due to differences in within-herd sampling rates. Furthermore, because herds represent finite populations, standard statistical methods for comparing proportions are not applicable to this type of survey data. A Monte-Carlo Bootstrap analysis was therefore developed to compare prevalence estimates across different herds groups. First, 1,000 Monte Carlo samples were generated by randomly sampling the within herd seroprevalence from the probability density function of the hypergeometric distribution, using, for each herd, the number of observed positive results, the number of sampled animals and the herd size as parameters. These Monte-Carlo samples allowed us accounting for the imprecision of within herd seroprevalence estimates. Then, following Johnston and Faulkner (15), a bootstrap comparison in median prevalence between two different herd groups was applied on each Monte-Carlo samples, resampling 5,000 times without replacement each time. Finally, a Monte Carlo *p*-value was computed across all Monte-Carlo bootstrapped samples. A Benjamini–Hochberg correction for multiple comparisons was applied when needed.

## 3 Results

### 3.1 EHDV morbidity in clinical outbreaks

In EHDV-8 outbreak farms, the number of clinical cases reported by farmers in cattle older than 24 months old, ranged between 1 to 30 leading to estimated morbidity rates from 0.5 to 33.9% (Q1 = 2.0%, median = 5.0%, mean = 8.0%, Q3 = 10.3%). Overall the morbidity rate was the highest in outbreaks from Zone 1 (median = 10.6%, range = 4.7–33.9%, *n* = 15), followed by outbreaks from Zone 2 (median 3.2%, range = 1.2–8.3%, *n* = 11), and the lowest in outbreaks from Zone 3 (median 0.7%, range = 0.5–1.2%, *n* = 4).

The clinical signs of EHD reported by farmers included hyperthermia, anorexia, hyperemia of the conjunctiva and the oral mucosa and/or ulceration of the oral cavity, muffle and nostrils associated with nasal discharge and lingual prolapse. Acute and chronic lameness were also frequently reported.

### 3.2 Prevalence of EHDV antibody positive cattle

Seropositive animals were detected in 26 of the 30 outbreak herds (86.6%) and in 24 of the 31 non-outbreak herds (77.4%). Eleven herds had no seropositive cattle, with two of these located in Zone 2 (1 outbreak and 1 non-outbreak) and the remaining seven in Zone 3 (3 outbreaks and 4 non-outbreaks).

In the four outbreak herds with no seropositive animals, only 1 to 2 clinical cases had been reported (morbidity between 0.5 and 1.2% in cattle over 24 months) with none of them still present at the time of blood sampling. In three out of eleven herds with an apparent null seroprevalence, all cattle over 24 months had been blood-sampled (22, 37, and 43 cattle, respectively). For the remaining eight herds, sampling rates were among the lowest (between 15.3 and 49.4%) due to large cattle numbers (between 81 and 317) and limited analyses (40 cattle tested except for one herd with 134 samples). Given these data, the upper bounds of confidence intervals for the estimated seroprevalences in these 11 herds ranged from 0 to 8.4%.

At the animal level, the overall prevalence of EHDV antibody positive cattle was significantly higher in animal from Zone 1 than in animals from Zones 2 and 3 (*p* < 10^−6^) (Table 2). Within Zone 1, the animal level seroprevalence was significantly higher in outbreak herds compared to non-outbreak ones (*p* < 10^−6^). This pattern was not observed in Zone 2 (*p* = 0.48) or Zone 3 (*p* = 1).

**Table 2:** Animal-level prevalence of EHDV antibody-positive cattle over 24 months of age from 61 outbreak and non-outbreak herds, stratified by geographical area, following the 2023 viral circulation in France.

Geographical zone	# positive/# tested	# cattle > 24 months old	Seroprevalence estimate (%) (exact 95%CI)
Zone 1			
Clinical outbreak	688/788	1,253	87.3 (85.7–88.7)
Non-outbreak	352/471	777	74.7 (72.1–77.2)
Overall	1,040/1,259	2030	82.6 (81.1–83.9)
Zone 2			
Clinical outbreak	51/473	524	10.8 (9.7–12.0)
Non-outbreak	59/473	621	12.5 (11.0–14.2)
Overall	110/946	1,145	11.6 (10.7–12.7)
Zone 3			
Clinical outbreak	1/252	717	0.4 (0.1–2.0)
Non-outbreak	1/305	866	0.3 (0.1–1.6)
Overall	2/557	1,583	0.3 (0.1–1.1)

**Figure 2:**
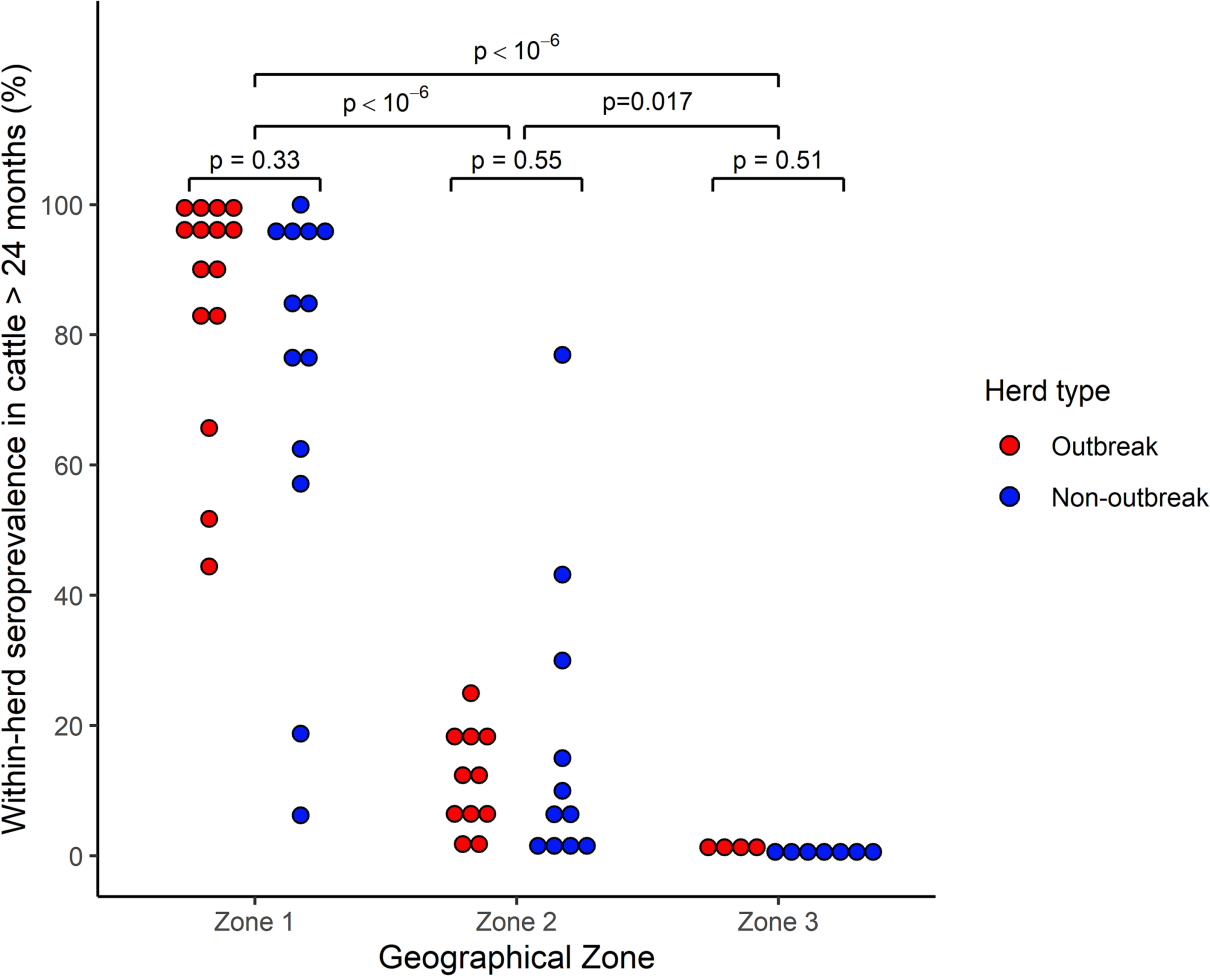
Distribution of within-herd prevalence of EHDV antibody-positive cattle over 24 months of age from 61 outbreak and non-outbreak herds, stratified by geographical area, following the 2023 viral circulation in France. Each point represents a herd. For ease of reading, only point estimates are represented, but confidence intervals could be wide given the heterogenous sampling rate across herds. Comparisons within and between Geographical zones were performed through a Monte-Carlo bootstrap comparison of medians.

However, this animal-level seroprevalence estimates do not account for the herd cluster effect and for the unequal sampling rate between herd. At the herd level, seroprevalence estimates were highly variable as shown in Table 3 and Figure 2. In all but 3 herds from Zone 1, the within-herd seroprevalence point estimate was above 50%, while this pattern was observed for only one herd in Zone 2. Notably, in Zone 1, point estimates exceeded 80% in 12 out of 15 outbreak herds (80.0%) and in 6 out of 7 non-outbreak herds (46.1%). In Zone 2, point estimates rarely exceeded 25%, except for 3 non-outbreak herds, while in the Zone 3, all point estimates were below 3%.

**Table 3:** Within-herd prevalence of EHDV antibody-positive cattle over 24 months of age from 61 outbreak and non-outbreak herds, stratified by geographical area, following the 2023 viral circulation in France.

Geographical zone	# herds	Within-herd seroprevalence point estimate (%)	
		Median	Range
Zone 1			
Clinical outbreak	15	94.7	44.4–100.0
Non-outbreak	13	83.3	6.2–100.0
Zone 2			
Clinical outbreak	11	11.9	0.0–25.0
Non-outbreak	11	7.5	0.0–76.9
Zone 3			
Clinical outbreak	4	0.0	0.0–2.6
Non-outbreak	7	0.0	0.0–1.2

Accounting for the uncertainty associated with variable sampling rates between herds, intra-herd seroprevalences were significantly higher in Zone 1 compared to both the Zone 2 and 3 (*p* = 10^−6^), but also higher in Zone 2 compared to Zone 3 (*p* = 0.017).

Conversely, within each geographical zone, herd-level seroprevalences did not differ significantly between outbreak and non-outbreak herds (Zone 1, *p* = 0.33; Zone 2, *p* = 0.55, Zone 3, *p* = 0.51).

### 3.3 Relationship between morbidity rate and within-herd seroprevalence

**Figure 3:**
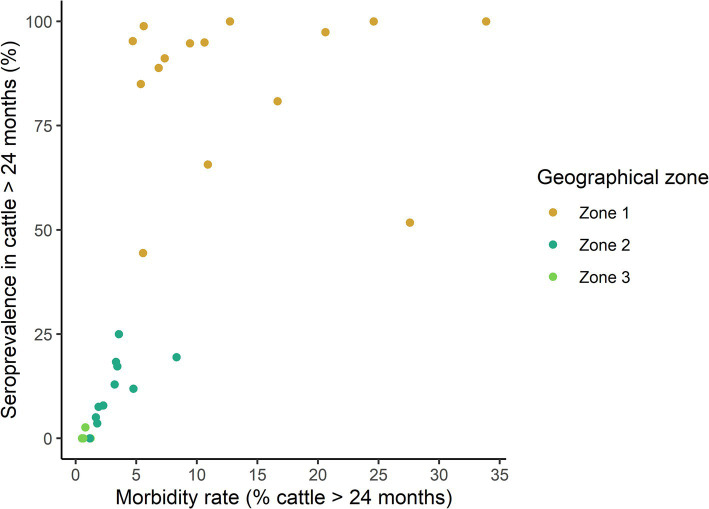
Relationship between the morbidity rate and the prevalence of EHDV antibody-positive cattle over 24 months of age from 30 clinical outbreak herds, stratified by geographical area, following the 2023 viral circulation in France. Each point represents a herd. For ease of reading, only seroprevalence point estimates are represented, but confidence intervals could be wide given the heterogenous sampling rate across herds.

To explore whether the morbidity rate could serve as a proxy for the within-herd level of immunity acquired after natural infection (as indicated by the within-herd seroprevalence), we examined the relationship between these two parameters in outbreak herds, accounting for the uncertainty of seroprevalence estimates. This correlation was strong across all investigated herds (Monte Carlo bootstrap median Spearman Rho = 0.85, *p* < 10^−6^), but was confounded by the major influence of the geographical zone (Figure 3). Specifically, morbidity rates between 5 and 10% were associated with markedly different seroprevalence levels in Zone 1 and 2. When focusing only on herds from Zone 1, this relationship was no longer significant (Monte Carlo bootstrap median Spearman Rho = 0.14, *p* = 0.6).

## 4 Discussion

To the best of our knowledge, this is the first study on within-herd seroprevalence of EHDV following the widespread circulation observed in France during the autumn of 2023. Our primary objective was to assess the variability of within-herd seroprevalence across three geographic regions that experienced clinical outbreaks to varying levels. To achieve this, our study design prioritized the inclusion of a large number of animals within each herd, enabling precise estimates of within-herd seroprevalence. This approach therefore differs from other studies, which aimed to estimate animal-level seroprevalence towards EHDV or BTV over broader geographic areas (16–19). Despite differences in study design, the animal-level seroprevalence estimates obtained in our study are in accordance with those obtained in other countries. In Zone 1, seroprevalence appeared very high and comparable to that observed in cattle or white-tailed deer in countries where EHDV is enzootic (19–21). Under the hypothesis of a reduced incidence of infection during the coldest months (from December 2023 to the end of May 2024), these high seroprevalence levels, reached in only 3 to 4 months in an initially naïve population, indicate very intense viral circulation and, therefore, high vector abundance and activity as well as a high susceptibility of cattle to EHDV-8.

Our results also highlight a strong south-to-north seroprevalence gradient, with a very low proportion of positive animals in Zone 3, the northernmost investigated zone. We acknowledge that the limited number of herds studied in this area may have hampered our ability to capture the full variability of seroprevalence levels and limit the statistical power. However, four of the seven clinical outbreaks officially recorded as of February 2024 in this area were included in our study, with similar results. This spatial variability is classically observed for vector-borne diseases and has been reported for both EHDV and BTV by several authors (19–22). Given the epizootic nature of EHDV-8 circulation in France between September and December 2023, the observed spatial heterogeneity was also likely influenced by temporal dynamics, with areas where the virus was detected earlier potentially exhibiting higher seroprevalence due to prolonged exposure to viral circulation. Indeed, although evidence of long-distance wind dispersion of *Culicoides* spp. has been shown for up to 700 and 500 km over sea and over land (23, 24), the local expansion of the infection zone occurs primarily from nearby areas. The first clinical cases of EHD reported in the Zone 2 (late October 2023) and Zone 3 (November 2023) occurred at a time when cooler temperatures were less favorable to vector activity and survival (11, 25, 26), and increased the extrinsic incubation period (11), thus reducing the incidence of new infections, even among animals in the same herd. This phenomenon probably partly explains the lower number of clinical outbreaks in these two northern zones, compared to Zone 1, and the lower seroprevalences observed in the investigated herds. The rare clinical outbreaks in Zone 3 could also be explained by the transport of clinically healthy but infected animals over long distances, despite the measures recommended for insect control, as already demonstrated for BTV (27).

Within-herd seroprevalence was generally very high in farms from Zone 1, regardless of whether they had experienced clinical cases or not. Although studies are warranted to precisely determine the duration of neutralizing antibodies against EHDV-8, studies on other EHDV variants or BTV suggest that immunity acquired after natural infection is likely to last for several years (13, 28). A high level of herd immunity is therefore expected in this zone. However, it should be noted that this post-infection immunity was heterogeneous, with some herds from Zone 1 having low to moderate seroprevalence levels. These observations may explain why new, albeit few, clinical cases have been observed in this area since the resumption of vector activity in June 2024, including in farms that had already reported clinical cases in 2023, as reported in other countries (22).

In the other two geographical areas studied, the low levels of collective natural immunity acquired after the 2023 viral circulation, even in herds that experienced clinical cases, suggest a high probability of a new epizootic when vector activity increases. These findings align with the many new clinical outbreaks detected since June 2024 in these regions (381 clinical outbreaks in the Loire-Atlantique department, as at December, 12, 2024).

The morbidity rate appeared highly variable among outbreak herds, as also recently reported in the same geographical area (10). Although the precise estimation of the morbidity rate was subject to caution in our study, as it depends on farmers’ ability to detect mild clinical signs and on observation conditions (e.g., animals housed indoors or grazing), the symptoms associated with the acute form of the disease are sufficiently pronounced to be readily noticeable. We therefore are confident that the risk of misclassifying herds as non-outbreak was low. EHDV and BTV infections produce indistinguishable clinical signs in cattle. At the time of our study, France was considered free of BTV-3 but BTV-8 had been reported in a few farms in Ariège (Zone 1), as well as in Tarn and Tarn-et-Garonne (Zone 2). However, none of the tested animals from the herds participating in our study were RT-PCR-positive for BTV-8. We cannot entirely rule out the presence of BTV-8-infected animals among clinically affected cows in some herds in these areas, since not all clinically ill individuals were tested in outbreak farms, and acknowledge a residual uncertainty. However, we believe any resulting bias is likely to be minimal. No clear relationship between the morbidity rate and seroprevalence could be established in outbreak herds. Notably, high seroprevalence levels were also observed in herds where farmers reported no affected animals. This very high heterogeneity in morbidity rate is probably linked to numerous factors (variability in the abundance of vectors, exposure, general condition and resilience of cattle…) that are difficult to objectively quantify at present. The challenging farming conditions in the autumn of 2023 (severe summer drought, poor forage quality and high parasite burden) may have influenced the animals’ resilience and variably contributed to the clinical expression in some animals across different herds.

The three geographical areas investigated were defined based on the timing and magnitude of EHD clinical outbreak incidence, but they may also differ in environmental factors that could significantly influence EHDV transmission dynamics, such as climate, landscape, vector ecology, farming practices or abundance of wild ruminants and domestic small ruminants. Other domestic or wild ruminants species could indeed serve as potential reservoirs of EHDV and aggravate the epidemiological cycle of EHD. EHDV-8 infection has been reported in clinically affected European red deer (*Cervus elaphus*) in Spain (9) following the 2022 epidemic; however, the serosurvey conducted by the same authors indicated limited spread of EHDV-8 among wild cervid populations in the country. In France, four spleens, collected from dead Pyrenean chamois (*Rupicapra pyrenaica*), roe or red deer, were found positive when tested by EHDV specific RT-qPCR, with high viral loads, suggesting recent EHDV infection in these animals (10). Surveillance of mortality rates in wildlife in Spain and South West of France, however, suggests a low impact of EHD. Similarly, only very few EHDV-8 positive cases were reported in sheep in Zone1 and 2, where sheep farms are numerous. Whether sheep and wildlife can play a significant role in EHDV-8 transmission dynamics in France remains uncertain (29), and deserve further investigation. Investigating such factors was however beyond the scope of our study, which aimed primarily to document the heterogeneous situation observed at the end of the 2023 epidemic in affected areas.

Despite early recommendations from health authorities at the onset of the epidemic to protect livestock against vectors through the use of chemical repellents, seroprevalence appeared to be very high in most herds in Zone 1, indicating extensive viral circulation in this area. Such findings have also been reported in white-tailed deer (*Odocoileus virginianus*) in Florida, with higher seroprevalence levels against EHDV in farmed deer undergoing aggressive chemical vector control compared to wild deer, suggesting that livestock densities may play a crucial role in the infection rate (30) and a poor efficacy of chemical repellents in face of abundant vectors populations (31–33). Conversely, grazing, which is associated with lower animal densities than indoor housing, was associated with higher risk of Bluetongue virus serotype 8 (BTV-8) infection in cattle in the Netherlands (34). In our study, nearly all farms were beef cattle farms, with grazing being the predominant practice. However, specific farming practices were not investigated, preventing the investigating of potential risk factors for EHDV infection.

From 2006 to 2009, France experienced major epidemics of both BTV-8 and BTV-1. Two years of mandatory vaccination against both serotypes in French mainland, followed by two additional years of voluntary vaccination campaigns, together with the high proportion of naturally infected animals led to a dramatic decrease in outbreaks (16). Consequently, mainland France was declared BTV-free again by December 2012. Similar vaccination measures could be effective against EHDV. The availability of an inactivated vaccine against EHDV-8 (35) since August 2024 presents a significant opportunity to control this new epidemic in Europe. In France, vaccination against EHDV-8 was initiated on September 23, on a voluntary basis, with the vaccine provided free of charge by the French State, within a North-West to South-East belt to prevent the disease’s further eastward spread. Mainland France also faces both a new-variant BTV-8 and BTV-3 epidemics since august of 2023 and 2024, respectively, with vaccines also available (10). However, with these three vector-borne diseases posing a significant threat to the livestock industry, crucial questions arise regarding vaccine interactions and the optimization of vaccination schedules to ensure the highest level of protection before vector activity resumes in late spring 2025.

## Data Availability

The raw data supporting the conclusions of this article will be made available by the authors, without undue reservation.
